# Optimal Design of Clinical Trials of Dietary Interventions in Disorders of Gut-Brain Interaction

**DOI:** 10.14309/ajg.0000000000001732

**Published:** 2022-03-16

**Authors:** Heidi M. Staudacher, Chu Kion Yao, William D. Chey, Kevin Whelan

**Affiliations:** 1 Deakin University, IMPACT (Institute for Mental and Physical Health and Clinical Translation), Food & Mood Centre, Geelong, VIC, Australia;; 2 Monash University and Alfred Health, Melbourne, Victoria, Australia;; 3 Division of Gastroenterology, Michigan Medicine, Ann Arbor, Michigan, USA;; 4 Department of Nutritional Sciences, King's College London, London, United Kingdom.

## Abstract

There is accumulating evidence for the fundamental role of diet in the integrated care of disorders of gut-brain interaction. Food is a complex mixture of components with individual, synergistic, and antagonistic effects, compared with the relative purity of a pharmaceutical. Food is also an inherent part of individuals' daily lives, and food choice is strongly tied to food preferences, personal beliefs, cultural and religious practices, and economic status, which can influence its ability to function as a therapeutic intervention. Hence, randomized controlled trials of dietary interventions carry unique methodological complexities that are not applicable to pharmaceutical trials that if disregarded can pose significant risk to trial quality. The challenges of designing and delivering the dietary intervention depend on the type of intervention (i.e., nutrient vs food supplementation or whole-diet intervention). Furthermore, there are multiple modes of delivery of dietary interventions, each with their own advantages (e.g., the high precision of feeding trials and the strong clinical applicability of dietary counseling trials). Randomized placebo-controlled trials of dietary interventions are possible with sufficient attention to their design and methodological nuances. Collaboration with experts in nutrition and dietetics is essential for the planning phase; however, even with expert input, not all challenges can be overcome. Researchers undertaking future dietary trials must be transparent in reporting these challenges and approaches for overcoming them. This review aims to provide guiding principles and recommendations for addressing these challenges to facilitate the conduct and reporting of high-quality trials that inform and improve clinical practice.

## INTRODUCTION

There is rapidly accumulating evidence for the importance of diet in the integrated care of disorders of gut-brain interaction (DGBI). Although there is substantial guidance from regulatory bodies for the design and reporting of pharmacological trials, there is no such guidance for dietary intervention trials, which are characterized by many unique complexities that if not properly accounted for can pose risks to trial quality and interpretation.

Drugs invariably contain a highly purified chemical compound given in a precise dose and formulated to increase the likelihood of delivery to the desired region of the gastrointestinal (GI) tract. Decisions on dose, frequency, and timing of administration are informed by pharmacodynamic, pharmacokinetic, and basic toxicity studies. These observations regarding drug delivery are in contrast to studies addressing the efficacy of diet as an intervention. Food is a complex amalgam of components with individual, synergistic, and antagonistic effects on the luminal microenvironment. The types and doses of nutrients and chemicals within food are also more variable than would be tolerated in a pharmaceutical. There are unique trial design challenges that come with dietary trials. In particular, how the diet intervention is delivered to study participants and the choice of comparator and blinding can all introduce bias that can reduce confidence in the results.

Given the rapidly progressing evidence for diet in the treatment of DGBI, an update of previous guidelines (1) is warranted. This review will define and describe the various types of dietary interventions and their mode of delivery, discuss the design and methodological complexities specific to dietary trials, provide new advice for measuring patient-centered end points, and provide recommendations for researchers to address these challenges to conduct methodologically rigorous, high-quality trials that can inform and improve clinical practice.

### Dietary intervention trials

Dietary interventions can involve supplementation with nutrients (“nutrient supplementation”) or foods (“food supplementation”), changes in whole diet (“whole diet intervention”), or restriction and rechallenge of specific dietary components (Table 1). Trials of restriction and rechallenge come with unique challenges (e.g., nocebo and physiological responses to nonfood challenges) that are described in detail elsewhere (1). For the purposes of this review, all classes of interventions will be referred to as “dietary interventions.” The mode of delivery of dietary intervention will affect the challenges and strengths and limitations faced.

**Table 1.**
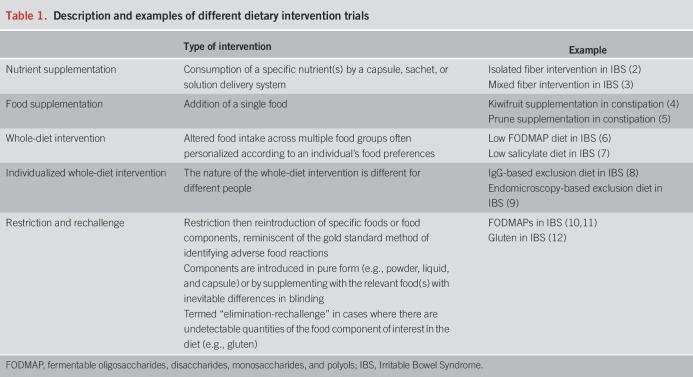
Description and examples of different dietary intervention trials

### Modes of delivery of dietary intervention

**Figure 1.**
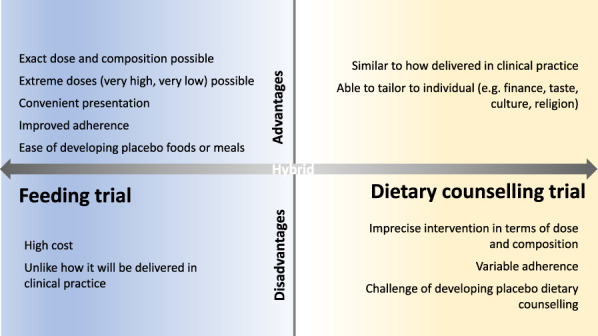
Advantages and disadvantages of delivering a dietary intervention as a feeding or dietary counseling trial. Feeding trials offer high precision in delivery of the intervention but are usually intensive and costly, whereas dietary counseling trials have greater external validity because they better resemble how an intervention is delivered in practice, although developing placebo dietary counseling is challenging. Hybrid models that combine feeding and dietary counseling are also used, whereby some foods/meals are provided and ad libitum intake is adjusted through dietary counseling.

Dietary interventions with foods or whole diets can be delivered either through direct feeding or dietary counseling or a hybrid of the 2, and there are advantages and disadvantages for each (Figure 1). Feeding trials involve provision of all food (and sometimes beverages) to participants for the duration of the intervention. Diets are carefully designed to alter only the dietary component(s) of interest while nutritionally matching all other aspects with the control group. Feeding trials can be performed either as a domiciled feeding trial, where participants reside at the research facility and researchers supply all dietary intake and measure outcomes (13), or in the free-living context, where meals are prepared in bulk and delivered fresh or frozen to participants. Hybrid feeding trials are also possible, whereby some meals (usually during working hours) are consumed at the research unit and the remainder (usually evening and weekends) are consumed at home (14). Web-based or mobile applications, while mostly used as an adjunct to dietary counseling, may also be used as the sole delivery method for whole diet interventions (15). Although trials are scarce, if these methods are shown to be safe and efficacious, this could enable widespread clinical use.

There are many major advantages of feeding trials including high adherence and the ability to design a bespoke placebo diet; however, feeding trials have several disadvantages (Figure 1). They are expensive, and external validity can be limited due to the inherent absence of challenges that jeopardize adherence when the dietary intervention is used in real life (e.g., participant motivation and understanding of advice provided).

Dietary counseling is the most common method of diet delivery in DGBI dietary trials and is highly applicable to the clinical setting. Participants are counseled as to the foods to include and/or restrict, and written resources are often provided. Counseling should be performed by a specialist gastroenterology dietitian who has the expertise to personalize the diet based on personal requirements (e.g., food preferences, cultural and religious practices, and socioeconomic restrictions) while ensuring nutritional adequacy. Explanation of the physiological effects of a diet may be provided in unblinded trials. There is inevitable imprecision in the intervention because each participant will follow a different diet and adhere to the intervention to differing degrees (Figure 1). Other dietary education modalities are also available, including group counseling (16,17), instruction provided through written text (e.g., books and online resources) or mobile applications. Education through a mobile application may be effective at improving symptoms (18), but evidence of optimal education strategies, or combinations of these, is limited.

### Design and methodological issues and recommendations for dietary trials

RCTs are the gold standard for determining the efficacy of a dietary intervention. Dietary RCTs can be designed as a parallel or crossover trial. Crossover trials require fewer participants but are more influenced by dropouts, and the minimum washout duration is often unclear. Run-in periods are sometimes used before randomization to homogenize or optimize background diet or to allow for adaptation (e.g., to high-fiber diet). Run-in periods should not be used generally as a method to select highly adherent participants because this leads to a highly selected population with limited real-world translation; however, this may be useful for feeding trials that are not designed to be pragmatic and in which minimizing attrition reduces the cost of running the trial. Regarding clinical end points, investigators should be encouraged to use high-quality, clinically or physiologically relevant, and validated end points from the literature.

There are additional methodological considerations for dietary trials, specifically in DGBI. In particular, trials including microbiome end points must consider the rapidity of diet-induced microbiome changes and the length of time required for diet-induced microbiome changes to stabilize in a new state of ecological homeostasis (19,20), the substantial temporal intraindividual variability in microbiome composition and function (21), and the potential confounding imparted by medication and comorbid conditions (22,23). A comprehensive summary of methodological issues relating to diet-microbiome research is provided elsewhere (24). Furthermore, just as pharmacokinetics inform design of pharmaceutical studies, assessment of physiological responses to diet relevant in DGBI, such as transit time and fermentation, should occur in consideration of the temporal effect of diet and the sampling conditions (e.g., fed or fasted state, habitual diet, and caloric content).

### Biases in dietary trials

**Figure 2.**
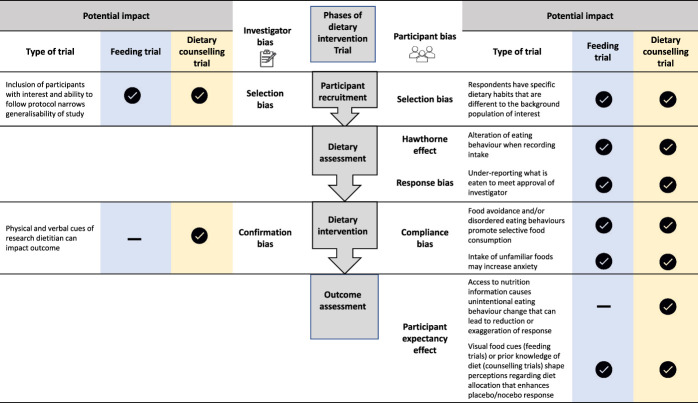
Potential for bias in dietary intervention trials. Some biases are unique to dietary intervention trials, and others are common to dietary, pharmaceutical, and behavioral trials. Tick icons indicate that the bias is present, and hyphens indicate that the bias is not present.

Biases in RCTs can be participant-derived or investigator-derived (Figure 2). Selection bias can alter baseline participant characteristics and subsequently interfere with accurate interpretation of clinical effects (25). For example, participants in a fiber supplementation trial with optimal habitual fiber intake may have differential responses to those with suboptimal fiber intake (26). Recruitment strategies that target individuals with a wide range of dietary habits and the use of a neutral language to mask the study hypothesis can help to mitigate selection bias.

The Hawthorne effect (modification of behavior in response to being observed) is common in both dietary trials and in DGBI. Both this and response bias can reduce accuracy of the dietary assessment, which can lead to underestimating energy and total fat intake (27). Strategies to reduce the impact of these biases are discussed below. Compliance bias can pose a considerable challenge during the intervention phase. Preexisting diet beliefs and behaviors can substantially influence an individual's ability to comply in dietary trials. A myriad of factors affect these behaviors including cultural background and ethical and religious beliefs (28), the high prevalence of perceived diet-symptom associations (29,30), and disordered eating (e.g., orthorexia nervosa and avoidant restrictive food intake disorder) (31) in DGBI. Excluding participants with disordered eating patterns may reduce the impact of this type of bias and mitigate safety concerns, particularly for whole-diet intervention trials.

Finally, expectations regarding the therapeutic potential of a food or diet based on prior knowledge can shape clinical responses to the dietary intervention or placebo and lead to participant expectancy effects. The increasing accessibility to diet information through lay media or the scientific community has facilitated widespread self-directed dietary experimentation, particularly in irritable bowel syndrome (IBS) (32), which could reduce or exaggerate response among participants. In whole-diet counseling trials, it may be necessary to include only intervention-naïve participants and/or to restrict information regarding the nature of the intervention where it is ethical and practical to do so.

### Intervention and placebo design and delivery

#### Precision of the intervention.

An inherent challenge of dietary trials is that excellent precision is not universally possible. The exact dose and composition of the intervention is not always known or able to be applied consistently across participants. Precision is influenced by the mode of delivery, adherence, and the degree of dietary confounding (Table 2). For example, nutrient supplementation trials with high levels of intervention “purity” (i.e., known dose and composition) achieve high precision because adherence is usually very good and can be monitored with accuracy, and the intervention rarely affects background dietary intake. Food supplementation trials are more subject to dietary confounding because the supplemented foods can lead to homeostatic displacement of other food(s). For example, 2 kiwifruits provide up to 25% of the daily fiber requirements and will affect intake of other fruits and snacks. Thus, changing one component of the diet leads to compensatory changes in other components, a problem termed dietary collinearity. The precision of whole-diet trials is highly dependent on the mode of delivery. Whole-diet feeding trials allow quantification and compensation for collinearity. However, whole-diet counseling trials usually incorporate personalized advice, which leads to dietary changes that differ between participants with variable collinearity influences on background diet. This must be weighed against the high clinical applicability and relatively low cost of dietary counseling to deliver the intervention.

**Table 2.**
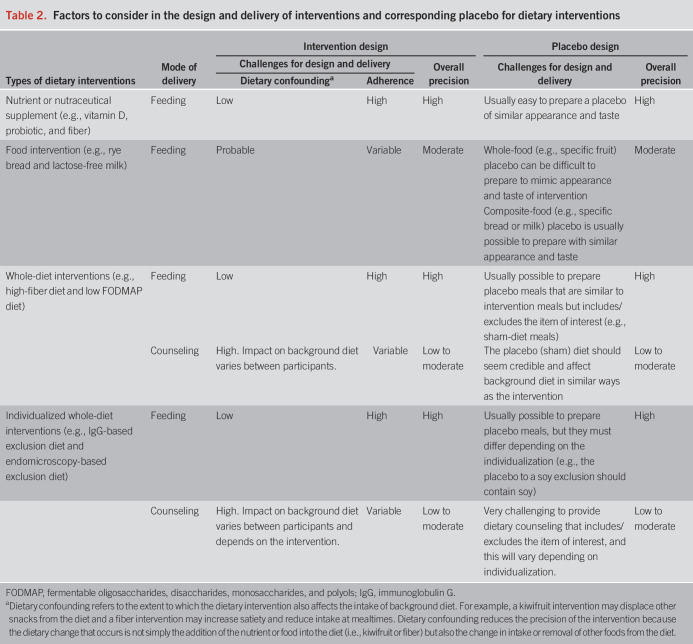
Factors to consider in the design and delivery of interventions and corresponding placebo for dietary interventions

##### Controls and blinding.

A unique complexity of dietary trials, and particularly whole-diet trials, is the selection and design of an optimal control. A host of options are available including habitual diet (inevitably unblinded) or “active” interventions such as an alternative diet or nondiet intervention. Placebo controls are, however, the gold standard choice because they facilitate treatment blinding. This is of extreme importance in DGBI for which end points are largely self-reported. Placebos are relatively straightforward to design in nutrient supplementation trials, and double blinding is easy to implement. However, incorporating a placebo control in food supplementation and whole-diet trials, in which the control must mimic the intervention to enable blinding but be inert, is more difficult (Table 2). The ease of designing a placebo control for a food depends on the nature of the component under investigation. This can be relatively easy (e.g., for caffeine, standard coffee vs decaffeinated coffee) or be complex and impossible (e.g., what makes a placebo for an apple?).

In whole-diet feeding trials, placebo controls can be delivered with high precision. Sham meals can be prepared to appear and taste similar to the intervention, and double blinding is possible because study meals can be double-coded by external staff before delivery (7,33). Implementing a placebo control for whole-diet counseling interventions is considerably more challenging (Table 2) and has been undertaken with varying degrees of rigor (8,34–37). A sham diet for this purpose must lead to similar complexities of dietary change but must not affect intake of nutrients or the food component of interest. Further criteria for sham diet design and testing are detailed elsewhere (38). The dietitian delivering the advice cannot be blinded (single-blind only); however, it is possible for the research team measuring outcomes to be blinded (double-blind). However, care must be taken to limit unblinding of the participants where interventions are accepted in practice (e.g., disguise the intervention in trial advertisements and only include participants who are diet-naïve).

In all cases, it is recommended that attempts to limit investigator bias where double blinding is not possible is described and that researchers collecting and assessing outcome data are blinded to participant allocation until analysis is final (39). The success of blinding should be measured (asking participants to guess their allocation at the end of the trial) and reported, although it can be influenced by whether the symptomatic response was achieved. Successful blinding might be indicated by high uncertainty about allocation or an equal distribution of correct and incorrect responses (40).

### Powering and sample size calculation

Like all clinical trials in DGBI, dietary trials should be adequately powered to detect differences in the primary outcome and should be accompanied by a sample size calculation. However, the availability of data required to calculate a sample size (i.e., effect size estimates for intervention and placebo and variability) presents unique challenges.

First, owing to the paucity of trials, data for the effectiveness of an identical dietary intervention or identical placebo on the primary outcome may not be available, and where available, they may not be measured in the same study.

Second, variation in response to dietary intervention is notoriously high resulting in high SDs for sample size calculations. For example, some people respond completely, some moderately, and some not at all, which is governed by background diet, physiological variation in the mechanisms of action of the intervention, and variability in adherence.

Third, placebo and nocebo effects for dietary interventions are high due to strong beliefs about diet and the often-intensive clinician-patient interaction. Although the high placebo effect in dietary trials replicates what occurs in real life, in research, it requires the dietary intervention to achieve a very high level of success to demonstrate efficacy over placebo that might approach the ceiling of response rates.

Overall, unknown or inconsistent data for the effect size of a dietary intervention and wide variation in response, partnered with high placebo rates, can result in calculated sample sizes that are prohibitively high and might exceed the available funding to complete high-quality dietary trials.

### Measuring dietary intake

Collection of dietary data enables objective evaluation of participant adherence in dietary trials. Adherence, at least in dietary counseling trials, can be influenced by skills of the dietary educator, participant understanding, health psychology, and financial and environmental access to foods. Adherence should ideally be determined by measuring intake of the dietary component of interest (e.g., fiber intake in g/d) rather than unvalidated arbitrary adherence measures (e.g., the threshold number of high-fiber foods consumed). Adequate adherence should be defined *a priori*. Dichotomous adherence ratings can be based on a specific threshold (e.g., 80% of nutrient/placebo supplements consumed (37) or 95% of study diet consumed (41)), for which there is no established gold standard or a statistically and nutritionally significant change in intake of the dietary component. Comprehensive dietary intake assessment also enables dietary confounding and nutritional adequacy to be measured and can enable derivation of measures including diet quality and dietary diversity (42), the latter recently performed using methods for determining microbiome alpha-diversity (e.g. Faith diversity) and beta-diversity (UniFrac distance metric (43)).

#### Methods of dietary assessment.

Dietary data can be obtained through prospective documentation (e.g., food records) or retrospective recall (e.g., food frequency questionnaire) of the type and quantity of food consumed (Table 3). Automated food and portion size identification through image-based technology can improve accuracy of data when used alongside traditional assessment methods (44), although it is not yet considered a suitable stand-alone tool (45). Mobile applications with barcode scanning facility enable rapid entry of dietary intake data, although it may not be suitable for granular analysis at the submacronutrient level (46). Dietary biomarkers, such as urinary metabolites, can be used as objective surrogate measures of recent intake (47), although application is currently limited by insufficient validation and high cost. The choice of prospective or retrospective methods of dietary data collection is determined by trial design, the dietary constituent(s) of interest, and resources available.

**Table 3.**
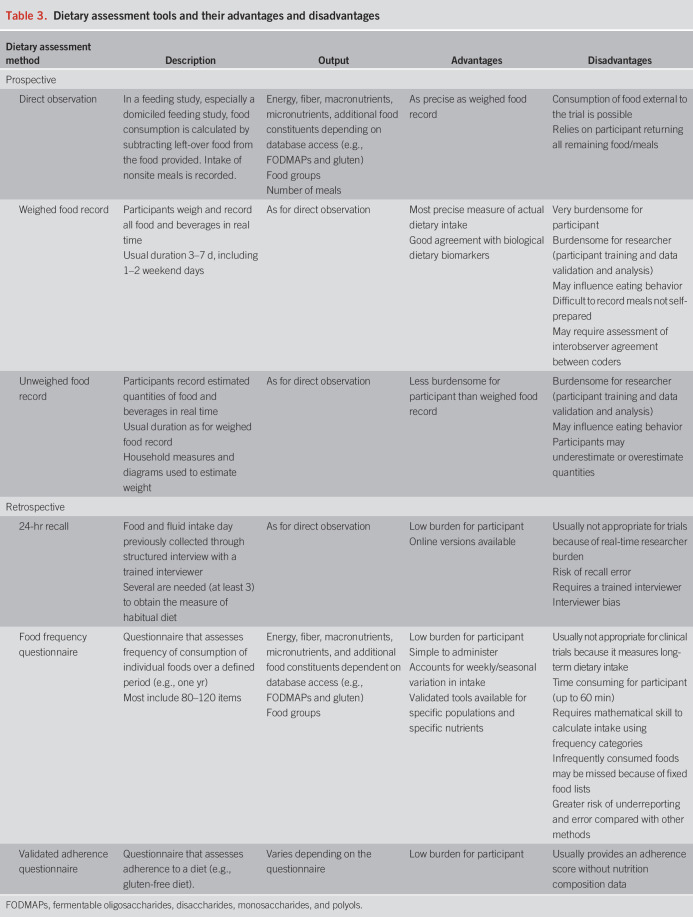
Dietary assessment tools and their advantages and disadvantages

#### Collecting high-quality dietary data.

One of the most acknowledged challenges of dietary research is the complexity of measuring diet. Accuracy of any dietary assessment method is limited because of the reporting error, Hawthorne effect and response bias (Figure 2), the systematic and random errors associated with coding of diet records, and the limitations associated with food composition data (48). Reporting error, at least with prospective methods, can be reduced by limiting recording duration. Most dietary trials use 3–7 days of recording, and 7 days has traditionally been deemed as sufficient in healthy individuals (49). For nutrients and foods where intakes have high within-subject coefficients of variation (i.e., eaten in widely variable amounts on different days), the number of days required to record actual intake with a high level of precision can be extreme (50). Recording duration for the greatest precision must be weighed against participant burden and the reduced accuracy resulting from “recording fatigue.”

The quality of dietary data can be optimized using several approaches. Provision of clear instructions to participants for completing dietary assessments and utilization of resources to enhance portion size accuracy (e.g., food models) will improve precision. Comprehensive cross-check of data should occur as soon as practically possible after data collection. Implausible data can be identified using calculations based on low and high energy intake cutoffs (51) or predicted energy requirements (52). Collaboration with a researcher in nutrition or dietetics is recommended.

### Adverse events, tolerability, acceptability, and quality of life

Although dietary therapies generally have a good safety profile compared with drugs, they may still carry some risk. Historically, dietary trials in DGBI have lacked standardized definitions of adverse events and many fail to report them at all. There has also been a focus on adverse events primarily consisting of worsened GI symptoms, with less frequent attention to extraintestinal manifestations, weight loss, or nutritional deficiencies. Adverse event data should be collected at prespecified intervals, and the reason for withdrawal should be recorded and reported.

Participation in a dietary trial can lead to unintended impacts on mental and social well-being, particularly in dietary counseling trials that mimic real-world practice. Therefore, tolerability, acceptability, and impact on food-related quality of life (FR-QoL) should also be assessed to fully evaluate the safety of a dietary intervention. Tolerability and acceptability are defined in various ways in the literature and are used interchangeably. Tolerability is commonly defined as GI tolerance but should encompass palatability, impact on hunger or satiety, and perception of food volume. Currently, there are no validated questionnaires for diet tolerability in DGBI. On the other hand, acceptability refers to satisfaction with integrating dietary changes into individuals' lifestyle encompassing domains such as accessibility, cost, convenience of meal preparation, and socializing. Studies have thus far used self-developed or self-adapted questionnaires that lack validation in GI disorders (41,53). A recently developed questionnaire has been partially validated to measure such domains of diet acceptability (54); however, this has yet to be used in DGBI.

FR-QoL is the extent to which the psychosocial roles of food, eating, and drinking bring enjoyment to peoples' lives (55). FR-QoL is worse in individuals with IBS who have more dietary restrictions (56); however, clinically effective therapeutic diets for DGBI may improve FR-QoL, but this has only been formally evaluated in the setting of a personalized low FODMAP diet for IBS (53). Instruments such as the FR-QoL-29 questionnaire (57) or Satisfaction with Food-related Life Scale (58), although only validated in other populations, are easy to administer and may be incorporated in dietary trials in DGBI. Altogether, data on adverse effects, tolerability, acceptability, and FR-QoL can help formulate recommendations for real-world practice regarding the safety and long-term suitability of the dietary intervention being evaluated.

### The research team

High-quality dietary research can only occur through interdisciplinary collaboration between clinicians, scientists, and bioinformaticians. Nuances of dietary research are best understood by skilled research dietitians or research nutritionists, who are increasingly undertaking senior roles in gastroenterology research. Dietitians or nutritionists should be funded, at least, to advise on trial design, deliver interventions (e.g., provide dietary counseling, design menus, and develop written resources), oversee nutritional safety, and collect and validate dietary intake data. For whole-diet trials in particular, the quality of the dietary counseling is highly dependent on the expertise of the dietitian. Statisticians familiar with statistical issues in dietary trials should also be consulted particularly for complex trial designs, sample size determination, and sensitivity analyses.

### Online resources

Digital tools, which can aid in the design and execution of dietary trials in individuals with DGBI, are increasingly available. A detailed accounting of these resources is beyond the scope of this review but can be found elsewhere (59). One high-value website for clinical investigators interested in dietary trials is Nutritools.org, which provides a comprehensive listing of validated dietary assessment tools, validation details, guidance on creating new tools, and links to food databases and data resources (e.g., guidance on data sharing). Other useful websites are given in Table 4.

**Table 4.**
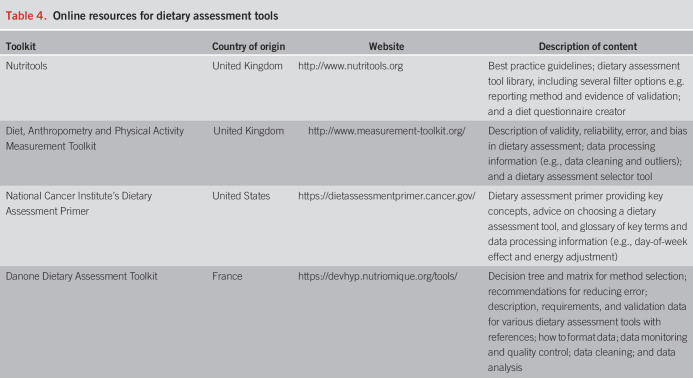
Online resources for dietary assessment tools

### Recommendations for reporting dietary trials

Recommendations have been prepared for reporting clinical trials of dietary interventions (Table 5). These recommendations were developed based on the experience of the authors and consulting the CONSORT 2010 guidelines for reporting randomized trials (60), the extended statement for nonpharmacologic treatments (39), the STROBE-nut extension for reporting nutritional epidemiology (61), the International Life Sciences Institute Europe Expert Group Guidelines for reporting studies to evaluate the health benefits of foods (62), and previous Rome Foundation Working Group Guidelines for the conduct of dietary trials in functional GI disorders (1). The intention is for these to be used in conjunction with the most appropriate reporting guideline (e.g., CONSORT). Those issues that cannot be overcome should be transparently reported. To do so will not only enhance the credibility of dietary research but also offer the greatest possibility of identifying evidence-based dietary treatment options.

**Table 5.**
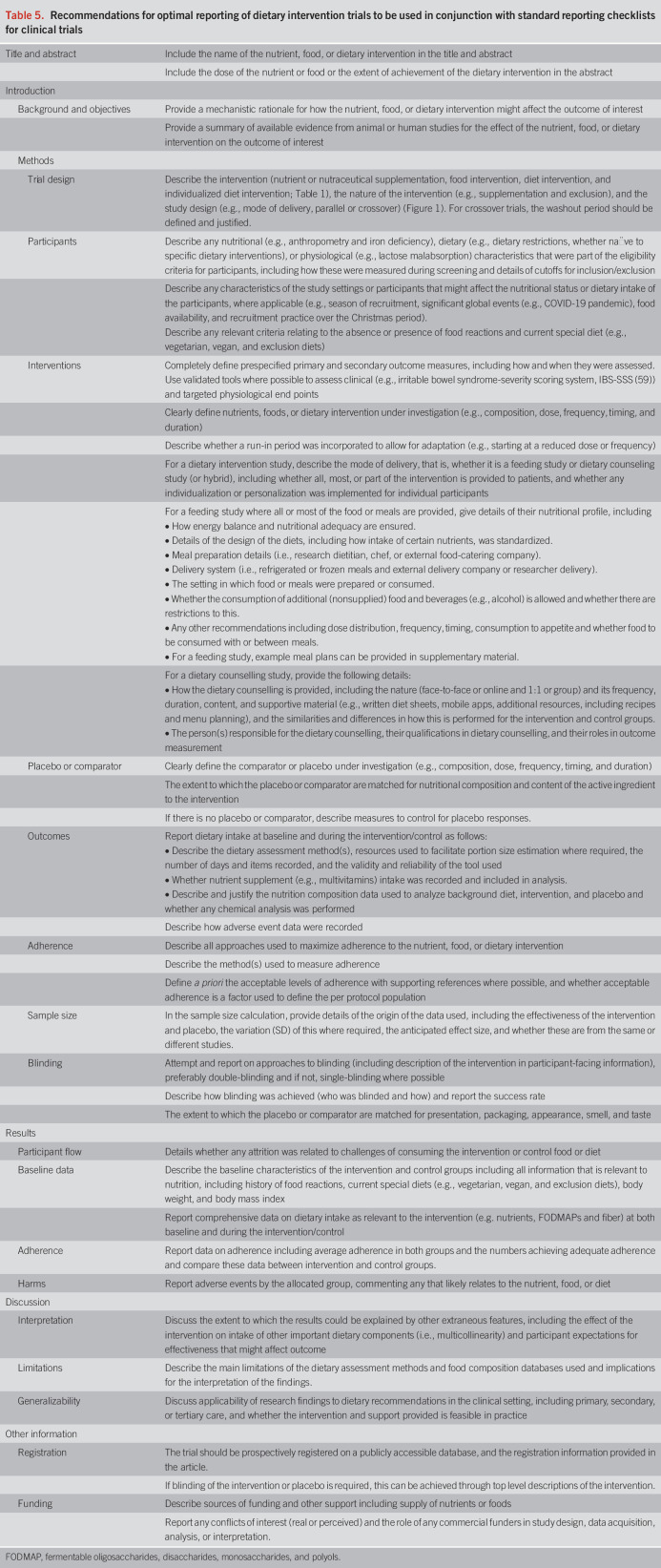
Recommendations for optimal reporting of dietary intervention trials to be used in conjunction with standard reporting checklists for clinical trials

## CONCLUSION AND FUTURE RESEARCH

Randomized placebo-controlled trials of dietary interventions are possible with sufficient attention to design and methodology nuances germane to dietary trials. Collaboration with experts in nutrition and dietetics is essential in the planning phase and for intervention delivery and the collection of high-quality dietary intake data and overseeing nutritional safety. Placebo controls and blinding are possible for nutrient supplementation, food supplementation and whole-diet interventions, and dietary confounding can be limited particularly in feeding trials. Biases are unavoidable in dietary trials but can be minimized and mitigated with appropriate strategies across recruitment and delivery of the intervention. Even with planning and support, some challenges cannot be overcome. Sample sizes required are often prohibitively high, and therefore, trials are regularly underpowered. Therefore, whether the same stringent criteria for assessing robustness of pharmacotherapy trials should apply to dietary trials for the purposes of evidence synthesis remains an issue for discussion. Researchers undertaking future dietary trials must be transparent in the reporting of design and methodology challenges and approaches used to overcoming them. Reporting of adverse events should be considered mandatory, and it is recommended that patient-centered outcomes such as tolerability, acceptability, and FR-QoL be measured for the safety evaluation of dietary interventions and therefore for informing their long-term suitability.

## CONFLICTS OF INTEREST

**Guarantor of the article:** Kevin Whelan, PhD.

**Specific author contributions:** All authors contributed to the conception of the article content; all authors contributed to drafting the article; H.M.S. collated the article; H.M.S. and K.W. edited the article; and all authors approved the final submitted article.

**Financial support:** None to report.

**Potential competing interests:** H.M.S. has received nonfinancial and financial support from CD Investments VSL Pharmaceuticals. C.K.Y. has received research funding from Crohn's Colitis Australia, Atmo Biosciences, and the International Organization for the Study of Inflammatory Bowel Disease. C.K.Y. works in a department that financially benefits from the sales of a digital application and booklets on the low FODMAP diet. Funds raised contribute to research of the Department of Gastroenterology and to the University. No author receives personal remuneration. W.D.C. is a consultant for AbbVie, Allakos, Alnylam, Bayer, Biomerica, Gemelli, Ironwood, Nestle, QOL Medical, Salix, Takeda, Urovant, and Vibrant and has research grants from NIH, FDA, Biomerica, Commonwealth Diagnostics, QOL Medical, and Salix. W.D.C. has stock options from GI on Demand/GastroGirl and Modify Health. KW receives research funding from the Almond Board of California, Danone, and the International Nut and Dried Fruit Council and is the coinventor of testing of VOC in the diagnosis and dietary management of IBS.
